# Prevalence and molecular characterization of *Cryptosporidium* spp. and *Giardia duodenalis* in deer in Henan and Jilin, China

**DOI:** 10.1186/s13071-018-2813-9

**Published:** 2018-04-12

**Authors:** Jianying Huang, Zhenjie Zhang, Yiqi Zhang, Yong Yang, Jinfeng Zhao, Rongjun Wang, Fuchun Jian, Changshen Ning, Wanyu Zhang, Longxian Zhang

**Affiliations:** 1 College of Animal Science and Veterinary Medicine, Henan Agricultural University, Zhengzhou, 450002 China; 2 Zhengzhou Foreign Language School, Zhengzhou, 450001 China; 3 College of Veterinary Medicine, Jilin University, Changchun, 130062 China; 4 Basic Medicine College of the Zhengzhou University, Zhengzhou, 450001 China

**Keywords:** *Cryptosporidium*, *Giardia duodenalis*, *SSU* rRNA, *gp60*

## Abstract

**Background:**

Little is known about the prevalence and zoonotic potential of *Cryptosporidium* spp. and *Giardia duodenalis* in deer in China. In this study, 662 fecal samples were collected from 11 farms in Henan and Jilin Provinces between July 2013 and August 2014, and were screened for the presence of *Cryptosporidium* and *G. duodenalis* with genotyping and subtyping methods.

**Results:**

*Cryptosporidium* spp. and *G. duodenalis* were detected in 6.80% (45/662) and 1.21% (5/662) of samples, respectively. Six *Cryptosporidium* species/genotypes were identified based on the small subunit ribosomal ribonucleic acid (*SSU* rRNA) gene: *C. parvum* (*n* = 11); *C. andersoni* (*n* = 5); *C. ubiquitum* (*n* = 3); *C. muris* (*n* = 1); *C. suis*-like (*n* = 1); and *Cryptosporidium* deer genotype (*n* = 24). When five of the 11 *C. parvum* isolates were subtyped by sequencing the 60 kDa glycoprotein (*gp60*) gene, zoonotic subtypes IIaA15G2R2 (*n* = 4) and IIdA19G1 (*n* = 1) were found. According to a subtype analysis, three *C. ubiquitum* isolates belonged to XIIa subtype 2. In contrast, only assemblage E was detected in the five *Giardia*-positive samples with small subunit ribosomal ribonucleic acid (*SSU* rRNA) gene sequencing.

**Conclusions:**

To our knowledge, this is the first study to report *C. andersoni*, as well as *C. parvum* zoonotic subtypes IIaA15G2R2 and IIdA19G1 in cervids. These data, though limited, suggest that cervids may be a source of zoonotic *Cryptosporidium* and *Giardia*. Cervids in the present study are likely to be of low zoonotic potential to humans, and more molecular epidemiological studies are required to clarify the prevalence and public health significance of *Cryptosporidium* and *G. duodenalis* in cervids throughout China.

## Background

*Cryptosporidium* and *Giardia* are two common protozoan parasites responsible for diarrhea in a broad range of vertebrate hosts, including humans, and domestic and wild animals worldwide. Transmission of both pathogens is by the fecal-oral route with both zoonotic and anthroponotic transmission cycles [1, 2]. The host plays an important role in the clinical impact of *Cryptosporidium* and *G. duodenalis* infections and the expression of disease. Drug treatments for these infections are inadequate, and do not provide a reliable strategy for their control [3].

Molecular epidemiological research into deer *Cryptosporidium* has been undertaken in red deer (*Cervus elaphus*), roe deer (*Capreolus capreolus*), swamp deer (*Rucervus duvaucelii*), sika deer (*Cervus nippon*), fallow deer (*Dama dama*), sambar deer (*Rusa unicolor*), caribou (*Rangifer tarandus*), white-tailed deer (*Odoileus virginianus*) and black-tailed deer (*Odocoileus hemionus*), in Europe (Spain, the Czech Republic and the UK), Asia (Nepal, Japan and China), Canada, the USA and Australia [4–18]. So far, 11 *Cryptosporidium* species/genotypes have been identified in cervids: *C. parvum*, *C. hominis*, *C. bovis*, *C. ryanae*, *C. ubiquitum*, *C. muris*, *Cryptosporidium* deer genotype, *Cryptosporidium suis*-like genotype, *Cryptosporidium* muskrat II genotype, *C. hominis*-like genotype and *Cryptosporidium* caribou genotype. *Giardia duodenalis* is considered a species complex that infects humans and many other mammals. Eight genetic groups or assemblages (A-H) have been identified based on a variety of genetic loci: assemblages A and B occur in humans and many other mammals; assemblages C and D in dogs; assemblage E in artiodactyls; assemblage F in cats; assemblage G in rodents; and assemblage H in seals [1]. However, only a few studies have reported the molecular characterization of *G. duodenalis* in cervids, with assemblages A, B, D and E identified [6, 13, 16, 19–29].

As an important center of mammalian evolution and dispersal, China possesses an abundance of deer species [30]. Deer and their products are of high economic value and deer farming has become an important component of China’s animal breeding industry. Sika deer, red deer, sambar deer, white-lipped deer, reindeer, Eld’s deer and Père David’s deer are the major species farmed in China. There are approximately 550,000 domesticated sika deer, most of which are distributed in northwestern China [31]. Velvet antlers, important in traditional Chinese medicine, are one of the main products derived from sika deer. Père David’s deer is an endemic species in China, but has become extinct in the wild [32].

Some epidemiological surveys exist concerning *Cryptosporidium* and *Giardia* in cervids around the world, but little is known about the prevalence and molecular characteristics of *Cryptosporidium* and *Giardia* in cervids in China. Only one study was conducted in Zhengzhou, where 124 fecal specimens were examined and two *C. ubiquitum* isolates were identified in sika deer [14]. In the present study, deer-derived *Cryptosporidium* and *Giardia* isolates were genetically characterized to better understand the distribution and zoonotic potential of the two pathogens in cervids in Henan and Jilin provinces.

## Methods

### Samples

In total, 662 samples were collected between July 2013 and August 2014 from 11 farms in Henan and Jilin provinces, from 16 red deer (*Cervus elaphus*), 47 Père David’s deer (*Elaphurus davidianus*) and 599 sika deer (*Cervus nippon*) (Table 1). Red deer and sika deer were in shed-feeding and housed in separate breeding houses according to different deer species and age groups. These 615 animals had a wide age distribution, ranging from 1 month to 15 years. However, the Père David’s deer from one forest farm were so agile, solitary and secretive, that it was difficult to determine their precise ages. Approximately 50 g of fresh feces was collected from each deer immediately after its defecation onto the ground, using a sterile disposal latex glove, and was then placed individually into a disposable plastic bag. No obvious clinical signs were observed in these deer, except for one case of diarrhea. The specimens were transported to the laboratory in an insulated container containing cold packs. Upon arrival, a portion of each specimen was examined by microscopy to detect *Cryptosporidium* oocysts and *Giardia* cysts using Sheather’s sugar flotation technique and Lugol’s iodine stain method, respectively. Wet smears were examined using a bright-field microscope with 100× and 400× magnification. All of the fecal specimens were stored in 2.5% potassium dichromate solution at 4 °C prior to DNA extraction.

**Table 1:** *Cryptosporidium* species/genotypes and *Giardia duodenalis* assemblages in cervids in this study

Province	Farm	No. of samples	*Cryptosporidium* spp.			*Giardia duodenalis*		
			No. positive	Prevalence (95% CI)	Species/genotype (*n*)	No. positive	Prevalence (95% CI)	Assemblage (*n*)
Jilin	Yutan-A	29	2	6.9 (0–16.7)	*C. parvum* (1); *C. ubiquitum* (1)	–	–	–
	Yutan-B	65	4	6.2 (0.2–12.2)	*C. andersoni* (1); *C. ubiquitum* (1); deer genotype (2)	–	–	–
	Shuangyang-A	52	6	11.6 (2.6–20.5)	*C. parvum* (2); *C. andersoni* (3); deer genotype (1)	–	–	–
	Shuangyang-B	32	2	6.3 (0–15.1)	Deer genotype (2)	–	–	–
	Zuojia-A	50	5	10 (1.4–18.6)	*C. parvum* (1); deer genotype (4)	–	–	–
	Zuojia-B	13	1	7.7 (0–24.5)	*C. parvum* (1)	–	–	–
	Zuojia-C	120	7	5.8 (1.6–10.1)	Deer genotype (7)	–	–	–
	Tonghua	102	5	4.9 (0.6–9.2)	*C. muris* (1); deer genotype (4)	–	–	–
Henan	Yuanyang	47	3	6.4 (0–13.6)	*C. ubiquitum* (1); deer genotype (2)	–	–	–
	Xinxian	56	5	8.9 (1.2–16.6)	*C. parvum* (2); *C. andersoni* (1); *C. suis*-like (1); deer genotype (1)	1	1.8 (0–5.4)	E (1)
	Qixian	96	5	5.2 (0.7–9.7)	*C. parvum* (4); deer genotype (1)	4	4.2 (0.1–8.2)	E (4)
Total		662	45	6.8 (4.9–8.7)	*C. parvum* (11); *C. andersoni* (5); *C. ubiquitum* (3); *C. muris* (1); *C. suis*-like (1); deer genotype (24)	5	0.8 (0.1–1.4)	E (5)

### DNA extraction

The fecal specimens were washed three times in distilled water and centrifuged at 3000× *g* for 10 min to remove the potassium dichromate. DNA was extracted from 200 mg of each fecal specimen using the E.Z.N.A. Stool DNA Kit (Omega Biotek Inc., Norcross, GA, USA), according to the manufacturer’s instructions. The extracted DNA was stored at -20 °C.

### Genotyping and subtyping

*Cryptosporidium* species were identified by nested PCR amplification and sequencing of an ~830 bp fragment of the small subunit ribosomal ribonucleic acid (*SSU* rRNA) gene, as described previously [2]. *Cryptosporidium parvum* and *C. ubiquitum* were subtyped by a sequence analysis of the 60 kDa glycoprotein (*gp60*) gene [33, 34]. The assemblages of *G. duodenalis* were determined by sequencing the small subunit ribosomal ribonucleic acid (*SSU* rRNA), β-giardin (*bg*), glutamate dehydrogenase (*gdh*), and triosephosphate isomerase (*tpi*) genes [35, 36]. Replicate analyses were done at each locus using both positive and negative controls.

### Sequence analysis

All PCR amplicons were sequenced on an ABI Prism™ 3730 XL DNA Analyzer (Applied Biosystems, Foster, CA, USA), using the BigDye Terminator v.3.1 Cycle Sequencing Kit (Applied Biosystems). The sequencing accuracy was confirmed by two-directional sequencing. The sequences were identified by their alignment with reference sequences downloaded from GenBank using the MEGA 6.0 software [37]. Representative nucleotide sequences generated in this study have been deposited in the GenBank database under the accession numbers KX259127-KX259145 and MG921620-MG921622.

### Statistical analysis

The statistical analysis was performed with the SPSS 22.0 software. Chi-square test and 95% confidence intervals (CIs) were used to compare the *Cryptosporidium* prevalence rates among different locations and age groups, and differences were considered significant at *P* < 0.05.

## Results

### Prevalence

Microscopic analysis of 662 cervine fecal samples showed an identical presence of *Cryptosporidium* oocysts and *Giardia* cysts to PCR assay. The overall prevalence of *Cryptosporidium* spp. in cervids was 6.8% (45/662, 95% CI: 4.9–8.7%). All 11 farms were positive for *Cryptosporidium*, with prevalences ranging between 4.9–11.5% (Table 1). No difference was observed in the *Cryptosporidium* prevalences in Jilin (6.91%, 32/463, 95% CI: 4.6–9.2%) and Henan (6.53%, 13/199, 95% CI: 3.1–10.0%) (*χ*^2^ = 0.032, *df* = 1, *P* > 0.05). Only five *Giardia*-positive samples were detected (0.76%, 5/662, 95% CI: 0.1–1.4%), from two farms in Henan Province (Table 1). *Cryptosporidium* was found in red deer, Père David’s deer, and sika deer, but *Giardia* was only detected in sika deer.

### *Cryptosporidium* species/genotypes

**Fig. 1:**
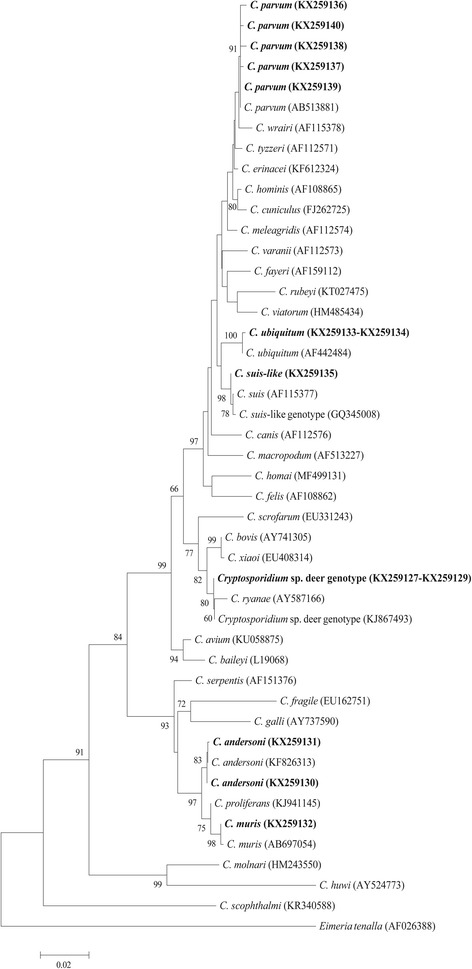
Evolutionary relationships among *Cryptosporidium* parasites inferred by Neighbour-joining analysis using the Kimura 2-parameter model based on the *SSU* rRNA gene sequences. Numbers on branches are percent bootstrap values from 1000 replicates. The newly generated sequences are indicated in bold

Forty-five *Cryptosporidium*-positive samples were genotyped by a sequence analysis of the *SSU* rRNA gene, and six *Cryptosporidium* species/genotypes were identified: *Cryptosporidium* deer genotype (*n* = 24, GenBank: KX259127-KX259129), *C. parvum* (*n* = 11, GenBank: KX259136-KX259140), *C. andersoni* (*n* = 5, GenBank: KX259130-KX259131), *C. ubiquitum* (*n* = 3, GenBank: KX259133-KX259134), *C. muris* (*n* = 1, GenBank: KX259132) and *C. suis*-like (*n* = 1, GenBank: KX259135). Phylogenetic relationship analysis confirmed the identity of *Cryptosporidium* species (Fig. 1). The *Cryptosporidium* deer genotype was the predominant genotype (*χ*^2^ = 7.901, *df* = 1, *P* < 0.001), and had an identical sequence to that isolated from white-tailed deer (KR260681) in the Czech Republic [10].

When the 11 *C. parvum* isolates sequences were compared with each other, five variants were detected. Variant 1 was identical to sequence AB513881 derived from calves in Egypt [38], while variants 2–5 had one or two nucleotide substitutions at six sites in the *SSU* rRNA sequence; these variants have only been found in this study. Two variants of *C. andersoni* were identified, and variant 1 and variant 2 were identical to sequences KF826313 and KF826314, respectively, isolated from Chinese outpatients with diarrhea [39].

### *Cryptosporidium* subtypes

All 11 *C. parvum* isolates were subtyped by sequence analysis of the *gp60* gene. However, only five of them produced the expected PCR products, which were identified as two subtypes: IIaA15G2R2 (*n* = 4, GenBank: KX259142) and IIdA19G1 (*n* = 1, GenBank: KX259141). IIaA15G2R2 was identical to strains isolated from cattle in the USA (DQ630517), and humans in Canada (DQ192501) and the USA (JX575583), while IIdA19G1 was identical to strains isolated from goats in China (KM199738), cattle in China (HQ009809) and Egypt (JX237824), and humans in China (JQ796092 and JF691561) and Sweden (KU852713). The *C. ubiquitum* isolates were subtyped to family XIIa and belonged to subtype 2 (GenBank: KX259143-KX259144), sharing 100% homology with a strain isolated from goats in China (KM199742) and a human-derived isolate from Turkey (JX412919). To the best of our knowledge, this is the first time IIaA15G2R2 and IIdA19G1 have been detected in deer.

### *Giardia* assemblage

**Fig. 2:**
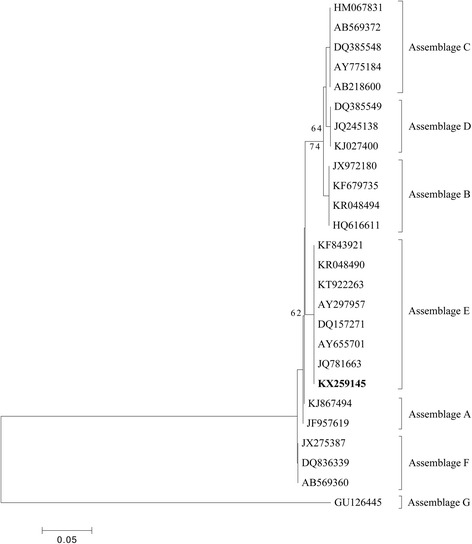
Evolutionary relationships among *Giardia duodenalis* inferred by Neighbour-joining analysis using the Kimura 2-parameter model of *SSU* rRNA gene sequences. Numbers on branches are percent bootstrap values from 1000 replicates. The newly generated sequence is indicated in bold

A total of five *G. duodenalis* isolates were successfully amplified and sequenced at the *SSU* rRNA (*n* = 5), *bg* (*n* = 3) and *gdh* (*n* = 2) loci, but amplifying failed at the *tpi* locus despite repeated attempts at molecular analysis using different primers. Sequence analysis showed that all the isolates belonged to *G. duodenalis* assemblage E. Comparison with *SSU* rRNA sequences available on GenBank showed 100% sequence identity with sequences of isolates previously recognized from calves (KT922263) and lambs (KT922264) in Ethiopia. Phylogenetic relationship analysis identified *G. duodenalis* assemblage E (Fig. 2). Two different subtypes were identified at the *bg* locus, which showed 100% similarity to strains isolated from sheep (GQ337972) in Norway and from calves (KT922247) in Ethiopia, respectively. At the *gdh* locus, the assemblage E shared 99% similarity with a yak isolate (KP334146) in China.

## Discussion

Results obtained from the 662 fecal specimens by both microscopic examination and PCR concurred. PCR is a tool of high sensitivity and specificity [40], particularly for identifying morphologically indistinguishable parasites such as species of *Cryptosporidium* and assemblages of *Giardia*, and for detecting their genetic variation. In contrast, Sheather’s sugar flotation technique and Lugol’s iodine staining are routine diagnostics. All the PCR-positive samples found positive by microscopy, and *vice versa*, may be due to sufficient oocyst and cyst concentrations in fecal specimens.

The *Cryptosporidium* prevalence of 6.8% in this study was close to the prevalence of 7.84% (25/319, 95% CI: 4.9–10.8%) reported in a study in Japan [9], but higher than that detected in Zhengzhou, China (1.61%, 2/124, 95% CI: 0–3.9%) [14]. It is difficult to explain the discrepancies in the prevalences of *Cryptosporidium* spp. among different studies because prevalences are affected by many factors, including the age distributions in the sample populations, sample sizes, management systems, seasons, examination methods and ecological conditions.

Studies of *Cryptosporidium* spp. in cervids have been conducted in several countries and 11 *Cryptosporidium* species/genotypes have been detected (Table 2). In this study, *Cryptosporidium* deer genotype was the most frequently detected. Small numbers of the *Cryptosporidium* deer genotype have been detected in red and roe deer in the UK [12, 15] and in white-tailed deer in the Czech Republic [10], whereas in sika deer in Japan [9] and white-tailed deer in the USA [13], *Cryptosporidium* deer genotype was the only genotype detected. It appears to be a host-adapted genotype, which has so far only been identified in deer.

**Table 2:** *Cryptosporidium* species/genotypes in cervids

Species/genotype	Host (Locality)	Reference
*C. parvum*	Red deer (UK, Czech Republic); roe deer (UK); white-tailed deer (USA); black-tailed deer (USA)	[4, 7, 11, 15]
*C. hominis*	Deer (Australia)	[16]
*C. bovis*	Roe deer (Spain)	[6]
*C. ryanae*	Roe deer (Spain); deer (Australia)	[6, 16]
*C. ubiquitum*	White-tailed deer (USA); roe deer (UK); swamp deer (Nepal); sika deer (China); red deer (Czech Republic); deer (Australia)	[5, 10–12, 14, 16]
*C. muris*	Red deer (Czech Republic); white-tailed deer (Czech Republic)	[10]
*Cryptosporidium* deer genotype	Red deer (UK); roe deer (UK); white-tailed deer (USA); white-tailed deer (Czech Republic); sika deer (Japan)	[9, 10, 12, 13, 15]
*Cryptosporidium* muskrat II genotype	White-tailed deer (USA)	[11]
*Cryptosporidium suis*-like genotype	Deer (Australia)	[16]
*Cryptosporidium* hominis-like genotype	White-tailed deer (USA)	[8]
*Cryptosporidium caribou* genotype	Caribou (Canada, USA)	[18]

*Cryptosporidium parvum* is one of the two most common *Cryptosporidium* species in humans [41]. We detected 11 *C. parvum* isolates, making *C. parvum* the second-largest cause of infection in cervids in this study. *Cryptosporidium parvum* infections were observed in red deer and roe deer in the UK [15], red deer in the Czech Republic [7], and white-tailed and black-tailed deer in the USA [4, 11]. We identified zoonotic subtypes IIaA15G2R2 and IIdA19G1 based on a sequence analysis of the *gp60* gene. Thus far, at least 14 *C. parvum* subtype families (IIa-IIi and IIk-IIo) have been found [2, 42]. IIa is the predominant subtype family in animals and humans worldwide, and IId is another major zoonotic subtype family reported in Europe, Asia, Egypt and Australia [43]. In China, most *C. parvum* isolates belong to subtype IId, including IIdA15G1 found in rodents, cattle and yaks [44–46], IIdA18G1 found in yaks [46], and IIdA19G1 found in cattle, humans, goats, yaks and urban wastewater [46–51]. In contrast, only a few IIa isolates have been detected in yaks and goats [48, 52], and IIc has been found in monkeys [53]. Subtype IIaA15G2R2 has previously been reported in humans, calves and water in the USA and Canada [54–56]. The above findings that the same *gp60* gene sequences of the *C. parvum* isolates have been found in humans and animals, suggest that deer infected with *C. parvum* in the areas we investigated might pose a threat to local people and animals by shedding oocysts in their feces, thereby contaminating the environment and food sources.

*Cryptosporidium ubiquitum*, previously called the *Cryptosporidium* cervine genotype, has been detected in sika deer in China [14], white-tailed deer in the USA [11], swamp deer in Nepal [5], roe deer in the UK [12], red deer in the Czech Republic [10] and deer in Australia [16]. In this study, three *C. ubiquitum* isolates were identified as zoonotic XIIa subtype 2, which has been found in domestic and wild ruminants, as well as in humans [34]. Despite its low prevalence in this study, *C. ubiquitum* may be a pathogen of public health concern in this area given its broad host range including rodents, carnivores, primates, domestic and wild ruminants, as well as humans in the UK, Slovenia, the USA, Canada, Spain and New Zealand [34].

Although *Cryptosporidium muris* is typically a parasite of mice and rats, it has a wide range of host species, including rodents, cats, marsupials (bilbies) and other mammals [57–59]. *Cryptosporidium muris* has also been identified in human cryptosporidiosis cases in many countries, including Thailand, Iran, India, Indonesia, Saudi Arabia, Kenya, Peru and France [60–67], and found in red and white-tailed deer in the Czech Republic [10]. *Cryptosporidium suis* naturally infects pigs worldwide, but has also been found in cattle, rodents, humans and chimpanzees [68], whereas the *C. suis*-like genotype has been reported in cattle and rodents, as well as in humans [12, 69, 70]. In this study, only one *C. muris* isolate and one *C. suis*-like isolate were identified. The susceptibility of deer to *C. muris* and *C. suis*-like is unclear due to inadequate data on these two *Cryptosporidium* species in cervids.

*Cryptosporidium andersoni* is predominantly detected in domestic cattle, although it has occasionally been found in other animals, including Bactrian camels, sheep and goats [71]. Only a few cases of human *C. andersoni* infection have been reported in France, Malawi, Iran, England and Australia, as well as in China [72]. Studies have reported 34 cases of *C. andersoni* infection in 252 human patients with diarrhea in Shanghai [73], and 21 out of 232 in Jiangsu Province [74]. To the best of our knowledge, this study is the first time *C. andersoni* has been confirmed in cervids. Five *C. andersoni* isolates were identified in deer, sharing 100% homology with strains isolated from outpatients with diarrhea in Jiangsu Province, China (KF826313 and KF826314) [74]. The source of *C. andersoni* infection and its transmission dynamics need further investigation to elucidate the zoonotic potential of *C. andersoni* in deer in China.

The prevalence of *G. duodenalis* was 0.76% in this survey, which is lower than that reported in fallow deer in Italy (11.5%) [21], red deer in Croatia (24.0%) [19], red deer, roe deer and moose in Poland (17.0–22.9%) [28], roe deer, reindeer and moose in Norway (7.1–15.5%) [75] and deer in Spain (5.4–8.9%) [6, 20, 76]. Globally, varying prevalence rates of *G. duodenalis* have been reported in fallow deer, red deer, roe deer, moose, caribou, reindeer, sambar deer and white-tailed deer in the USA, Canada, Australia, Croatia, Spain, Poland, Italy, the Netherlands, Norway and Sweden, ranging between 0.6–24.0% [6, 13, 16, 19–29, 75–78] (Table 3). In the PCR assay, all the samples were failed amplifying *Giardia tpi* region. A possible explanation for the result is that inhibitory problems existed in PCRs.

**Table 3:** *Giardia duodenalis* prevalences and genotypes in cervids

Location	Host	No. of samples	No. positive	Prevalence (95% CI)	No. of samples genotyped	*Giardia* spp./ *Giardia duodenalis* assemblage	Reference
Italy	Fallow deer	139	16	11.5 (6.1–16.9)	8	A-I (8)	[21]
Croatia	Red deer	374	4	1.1 (0.4–3.1)	4	A (3), D (1)	[19]
	Roe deer	21	5	24.0 (8.2–47.0)	5	A (2), D (2), *G. microti* (1)	
Poland	Red deer	61	1	1.6 (0–4.9)	1	A-III (1)	[22]
	Roe deer	50	2	4.0 (0–9.6)	2	A-I (2)	
Poland	Red deer	28	5	17.9 (2.7–33.0)	4	B (4)	[28]
	Roe deer	48	11	22.9 (10.6–35.2)	8	B (8)	
	Moose	23	4	17.0			
USA	White-tailed deer	80	1	1.3 (0–3.7)	1	A (1)	[13]
USA	White-tailed deer	26	1	3.8 (0–11.8)	1	A (1)	[23]
USA	White-tailed deer	394	5	1.3 (0.2–2.4)			[78]
USA	Reindeer				1	A (1)	[27]
Spain	Roe deer	212	19	8.9 (5.1–12.8)	7	A-II (7)	[6]
Spain	Roe deer	224	12	5.4 (2.4–8.3)			[20]
Spain	Deer	181	14	7.7 (3.8–11.7)			[76]
Sweden	Fallow deer				2	A (1), E (1)	[24]
	Moose				1	A (1)	
Italy	Fallow deer				8	A-III (8)	[25]
Norway	Reindeer				6	A (6)	[26]
	Moose				13	A (13)	
Norway	Red deer	289	5	1.7 (0.2–3.2)			[75]
	Roe deer	291	45	15.5 (11.1–19.6)			
	Reindeer	155	11	7.1 (3.0–11.2)			
	Moose	455	56	12.3 (9.3–15.3)			
Australia	Sambar deer, red deer, fallow deer	1563	10	0.6 (0.2–1.0)	10	A-I (1), A-III (9)	[16]
Netherlands	Roe deer				1	A (1)	[29]
Canada	Caribou	149	3	2.0 (0–4.3)			[77]

At the molecular level, this study is the first to characterize *G. duodenalis* from cervids in China, and only assemblage E was identified. Worldwide, less than 100 *G. duodenalis* isolates from cervids have been analyzed, among which assemblages A, B, D and E were detected [6, 13, 16, 19–29]. Assemblage A, including sub-assemblages A-I (infecting most animals), A-II (mainly found in humans), and A-III (mainly infecting wild ruminants), have previously been reported from red deer, roe deer, fallow deer, reindeer, white-tailed deer and moose in the USA, Croatia, Spain, Poland, Italy, Norway and Australia [6, 13, 16, 19, 21–27, 29]. Assemblage A is most frequently found assemblage in cervids, while assemblages B and D have only been found in red and roe deer in eastern Poland and Croatia, respectively [19, 28]. In Sweden assemblage E has been detected in a fallow deer [24]. *Giardia duodenalis* assemblage E has a wide distribution in domestic mammals, including cattle, water buffaloes, sheep, goats and pigs, and is the predominant assemblage found in these animals in the USA, Europe and Australia [1]. However, assemblage E has rarely been identified in wild hoofed animals, and thus may reflect an adaption to these animals following domestication [19]. Moreover, assemblage E has also been found in NHPs from western Uganda and China [79, 80] and in humans from Egypt, Brazil and Australia, suggesting zoonotic transmission of assemblage E [81–85]. Although assemblage E was detected in low numbers in this study, it is still important to understand the public health risk posed by the *Giardia* species and assemblages infection in cervids in the region.

## Conclusions

The 662 samples collected from red deer, Père David’s deer, and sika deer were screened for the presence of *Cryptosporidium* and *G. duodenalis*. *C. parvum*, *C. andersoni*, *C. ubiquitum*, *C. muris*, *Cryptosporidium suis*-like genotype, *Cryptosporidium* deer genotype and *G. duodenalis* assemblage E were detected in this study. This is the first study to report *C. andersoni*, as well as *C. parvum* zoonotic subtypes IIaA15G2R2 and IIdA19G1 in cervids. Deer farming has become an important component of China’s animal breeding industry. Farming increases the potential contact between deer and humans, and intensifies the numbers of animals, which potentially increases the numbers of shed (oo) cysts in the environment. Given that we have detected human pathogens in deer in China, further investigations into the transmission dynamics of these pathogens would be warranted.

## Abbreviations

*bg*: β-giardin
*gdh*: Glutamate dehydrogenase
*gp60*: 60 kDa glycoprotein
PCR: Polymerase chain reaction
*SSU* rRNA: Small subunit ribosomal ribonucleic acid
*tpi*: Triosephosphate isomerase
